# Supplementary material for: WordCommentsAnalyzer: A windows software tool for qualitative research
Source: F1000Res. 2018 Sep 4;7:536. Originally published 2018 May 3. [Version 2] doi: 10.12688/f1000research.14819.2 (PMC6137411; doi:10.12688/f1000research.14819.2)
Supplement: Supplementary file 3 [file f1000research-7-17614-s0002.tgz › b2a79496-4ada-47c4-9682-a99ce867836d.zip!/Eight Iranian Health Journals/Journal of Knowledge and Health_16.docx]

© Journal of Knowledge and Health. This work is licensed under CC BY 4.0 (https://creativecommons.org/licenses/by/4.0/).

Volume & Issue: دوره 9، شماره 2: 1393

Title: بررسی مقایسه‌ای تأثير ويتاگنوس و ویتامین E بر اضطراب زنان مبتلا‌به درد سینه دوره‌ای: كارآزمايي باليني تصادفی‌شده

http://knh.shmu.ac.ir/index.php/site/article/view/408

مقدمه: نزديكبهنيمميلياردنفردرسراسر جهاندچاراختلالات روانيبه‌خصوص اضطراب هستند. اختلالات اضطرابی در زنان شایع‌تر بوده و یکی از علل این شیوع، مسائل و مشکلات هورمونی است. هدف از این پژوهش بررسی تأثير درمان‌هاي شايع به‌منظور كاهش اختلالات هورمونی زنان بر ميزان اضطراب آنها است. مواد و روش‌ها: در این کارآزمایی بالینی 210 زن مبتلا به ماستالژی به‌روش نمونه‌گیری آسان انتخاب و به‌صورت تصادفی به سه گروه تقسیم شدند. در ابتدا سطح اضطراب به‌وسیله پرسشنامه بک سنجیده و سپس به‌مدت سه ماه به گروه اول داروی ویتاگنوس و به گروه دوم ویتامین E داده شد. در گروه سوم به‌عنوان شاهد هیچ مداخله‌ای انجام نشد. در پایان مجدداً امتیاز اضطراب سنجیده و با هم مورد مقایسه قرار گرفت. نتايج: میانگین نمره اضطراب بک در زمان ورود به مطالعه در گروه ویتاگنوس 4/2±6/24، در گروه ویتامین E، 4/1±7/22 و در گروه شاهد 3/3±3/23 بود و تفاوت آماری معنا‌داری بين اين گروه‌ها مشاهده نشد؛ در پایان پژوهش میانگین امتیاز اضطراب بک در سه گروه با هم تفاوت داشت و درگروه ویتاگنوس به 1/2±6/12 رسید که نسبت‌به گروه ویتامین E با ميانگين 1/1±5/19 و گروه شاهد با ميانگين 4/3±2/20 سطح اضطراب بیشتر کاهش یافته بود. نتیجه‌گیری: هر دو دارو باعث کاهش سطح اضطراب شدند؛ ولی این تأثير با مصرف ویتاگنوس بیشتر دیده شد. از آنجا که ویتاگنوس در بسیاری از بیماری‌های زنان مورد استفاده قرار مي‌گیرد و هزینه آن نیز پایین است؛ مي‌توان با استفاده از خواص ضد اضطراب این دارو، مصرف داروهای شیمیایی ضد اضطراب را کاهش داد.

Title: تأثیر مصرف مکمل زنجبیل و تمرینات استقامتی پیش‌رونده بر شاخص‌های آسیب سلولی در دانشجویان مرد غیرورزشکار

http://knh.shmu.ac.ir/index.php/site/article/view/58

مقدمه: هدف از این مطالعه بررسی تأثیر مصرف مکمل زنجبیل بر شاخص‌های آسیب سلولی ناشی از 6 هفته تمرینات استقامتی پیش‌رونده در دانشجویان مرد غیرورزشکار مي‌باشد. مواد و روش‌ها: 22 مرد غیرورزشکار (دانشجويان دانشگاه شاهرود) به‌صورت تصادفی در دو گروه مکمل (11 نفر) و دارونما (11 نفر) قرار گرفتند. سپس هر دو گروه به مدت 6 هفته، همزمان با مصرف مکمل زنجبیل یا دارونما به اجرای تمرینات استقامتی پیش‌رونده پرداختند. نمونه‌های خون قبل و بعد از دوره تمرینی و مکمل‌گیری  از آزمودنی‌ها گرفته شد و سپس غلظت پلاسمايي آنزیم‌های کراتین‌کیناز (CK) و لاکتات‌دهیدروژناز (LDH) به‌عنوان شاخص‌های آسیب سلولی اندازه‌گیری شد. نتايج: افزایش معنی‌داری در هر دو گروه دارونما و مکمل در فعالیت آنزیم‌های CK، LDH پس از 6 هفته تمرین استقامتی پیش‌رونده مشاهده شد همچنين مکمل زنجبیل نیز تأثیر معناداری بر فاکتور LDH در گروه مصرف‌كننده مکمل  نسبت‌به گروه مصرف‌كننده دارونما داشت نتیجه‌گیری: این نتایج نشان داد كه تمرینات استقامتی پیش‌رونده سبب افزایش فعالیت آنزیم‌های CK و LDH می‌شود و مصرف روزانه مکمل زنجبیل می‌تواند آسیب عضلانی ناشی از این نوع تمرینات را كاهش دهد.

Title: تأثير روان‌درمانی وجودی به شیوه گروهی بر رضایت زناشویی زنان مبتلا به دیابت نوع 2

http://knh.shmu.ac.ir/index.php/site/article/view/347

مقدمه: دیابت به‌عنوان یک بیماری مزمن تمام سطوح زندگی فردی و اجتماعی بیمار را تحت تأثير قرار می‌دهد. شیوع اختلالاتی از قبیل افسردگی، اضطراب، در اين بيماران و نيز عوارض جسمانی ناشی از این بیماری تعاملات خانوادگی و زناشویی را تحت تأثير قرار داده لذا انجام مداخلات در این رابطه ضروری به‌نظر می‌رسد.  هدف پژوهش حاضر تبیین تأثير روان‌درمانی وجودی به شیوه گروهی بر رضایت زناشویی زنان مبتلا به دیابت نوع 2 می‌باشد. مواد و روش‌ها: در پژوهش حاضر 16 زن عضو انجمن دیابت استان تهران به‌طور تصادفی انتخاب و در گروه آزمایش و کنترل قرار گرفتند (هر گروه 8 نفر). از هر دو گروه پیش‌آزمون به عمل آمد. گروه آزمایش در 8 جلسه رواندرمانی وجودی به‌صورت گروهی شرکت کردند، اما گروه کنترل مداخله‌ای دریافت نکردند. در پایان پس‌آزمون به‌عمل آمد و داده‌ها با روش تحلیل کوواریانس تحلیل شد. نتایج: نتایج نشان می‌دهد که بین میانگین نمرات پیش‌آزمون و پس‌آزمون گروه آزمایش تفاوت معناداری وجود دارد. این تفاوت در سطح 01/0 معنادار است. لذا می‌توان گفت روان‌درمانی وجودی به شیوه گروهی موجب افزایش رضایت زناشویی زنان مبتلا به دیابت نوع 2 شده‌است. نتیجه‌گیری: نتایج پژوهش حاضر نشان می‌دهد روان‌درمانی وجودی به شیوه گروهی می‌تواند موجب افزایش رضایت زناشویی زنان مبتلا به دیابت نوع 2 گردد. لذا انجام مداخلات بر اساس این رویکرد در رابطه با بیماران دیابتی منجر به بهبود زندگی زناشویی آنها می‌شود.

Title: بهينه‌سازي روش فنول کلروفرم سيليکا براي استخراج DNA از نمونه استخوان‌هاي قديمي

http://knh.shmu.ac.ir/index.php/site/article/view/350

مقدمه: استخراج DNA از نمونه استخوان‌هاي قديمي همواره با مشکلات زيادی همراه است. روش فنول کلروفرم سيليکا یکی از روش‌هائی است که به‌طور معمول برای این منظور استفاده مي‌شود. در اين مطالعه هدف بررسي و بهينه‌سازي مراحل این روش است. مواد و روش‌ها: DNA 62 نمونه استخوان (داراي عمر 3 تا 11 سال) ابتدا با روش فنول کلروفرم معمولي و سپس با تغيير بعضي پارامترها استخراج شد. DNA حاصل در هشت منطقه STR به نام‌هاي CD4, vWA, LPL, D5S818, D16S539, D13S317, F13, FES توسط واکنش PCR تکثير شد و نتايج حاصل برروي ژل اکريلاميد مقايسه شد. نتایج: ميانگين بازدهي واکنش PCR، براي روش جديد و روش معمولی در هشت منطقه پلي‌مورفيک ذکر شده به‌ترتيب 75%، 78%، 81%، 76%، 85%، 71%، 89%، 86%و 64%، 39%، 70%، 49%، 68%، 76%، 71%،28% بود.متوسط ميزان DNA حاصل از روش بهينه شده (در غلظت µl35 ذرات سيليکا) و معمولي به‌ترتیب برابر µg/ml 5/267 با خلوص 12/1 و µg/ml 76/192 با خلوص 84/0 بود. نتيجه‌گيري: طبق يافته‌هاي اين مطالعه به‌نظر مي‌رسد تيمار طولاني‌تر با EDTA يکي از عوامل مؤثر حذف بهتر کلسيم باشد و همچنين غلظت مناسب ذرات محلول سيليکا مي‌تواند تأثير مهمي در حذف مهار‌کننده‌هاي PCR داشته باشد.

Title: بررسی رابطه بین ویژگی‌های شخصیت و سلامت روان با رضایت شغلی

http://knh.shmu.ac.ir/index.php/site/article/view/392

مقدمه: پژوهش حاضر با هدف بررسی رابطه ویژگی‌های شخصیت و سلامت روان با رضایت شغلی کارکنان مراکز بهداشتی و درمانی انجام شد. مواد و روش‌ها: در یک مطالعه‌ی توصیفی- مقطعی 200 نفر از کارکنان دانشگاه علوم پزشکی شاهرود در سال 1390 به‌صورت تصادفی منظم انتخاب و پرسشنامه‌های ویژگی‌های شخصیتی نئواف‌اف‌آی، سلامت عمومی گلدبرگ و رضایت شغلی اسپکتور را تکمیل نمودند. تحليل داده‌ها با استفاده از آزمو‌ن‌های آماری توصیفی و ضریب همبستگی پیرسون و آزمون رگرسیون انجام شد. نتايج: يافته‌ها نشان داد که بین رضایت شغلی با عامل روان رنجوری رابطه منفی و با عامل برون‌گرایی رابطه مثبت معنا‌داری وجود دارد (05/0>P)، بین رضایت شغلی و سلامت روان رابطه منفی معنا‌داری وجود دارد (01/0P<)، هیچ یک از ویژگی‌های شخصیت نمی‌توانند رضایت شغلی را پیش‌بینی کنند اما تنها متغیر سلامت روانی می‌تواند رضایت شغلی افراد را پیش‌بینی کند. نتیجه‌گیری: به‌نظر می‌رسد افزایش حمایت شغلی و امکان مشاوره روانپزشکی و روان‌شناختی برای کارکنان در کنار بهبود تسهیلات و درآمد، مي‌تواند در ارتقای سلامت عمومی، افزایش رضایت شغلی آنان و درنتیجه بهبود کیفیت خدمات بهداشتی ارائه شده توسط آنها مؤثر باشد.

Title: ارزیابی صحت خودگزارش‌دهی مواجهه با دود سیگار در دوران بارداری از طریق اندازه‌گيري سطح کوتینین بندناف نوزاد

http://knh.shmu.ac.ir/index.php/site/article/view/454

مقدمه: مواجهه با دود محیطی سیگار در دوران بارداری منجر به عوارض جنینی و نوزادی مي‌گردد. از آنجایی‌که بررسی تأثير مواجهه با دود سیگار در دوران بارداری بر پایه خودگزارش‌دهی مادران ممکن است دقیق نباشد، لذا این مطالعه به‌منظور بررسی صحت گزارش مادر از مواجهه با دود سیگار با کوتینین سرم بندناف نوزاد در زمان زایمان انجام شده است. مواد و روش‌ها: مطالعه حاضر به‌صورت مقطعی برروي 108 خانم باردار غیرسیگاری در زمان زایمان انجام شد که براساس گزارش خودشان به دو گروه مواجهه‌یافته (54 نفر) و مواجهه نیافته (54 نفر) با دود سیگار تقسیم شدند. مواجهه با دود سیگار در دوران بارداری ابتدا از طریق پرسشنامه وضعیت مواجهه با دود سیگار و سپس از طریق اندازه‌گيري کوتینین سرم بندناف ارزیایی شد، بدین صورت که نمونه خون بندناف بعد از تولد نوزاد به‌منظور بررسی سطح کوتینین (متابولیت اصلی نیکوتین) در اتاق زایمان گرفته شد. جهت تخمین صحت خودگزارش‌دهی مادران از مواجهه با دود سیگار از نقطه برش کوتینین سرم بندناف نوزادان استفاده گردید که نقطه برش در این مطالعه بیش از 2 نانوگرم بر میلی‌ليتر به‌عنوان مواجهه یافته در نظر گرفته شد. نتايج: میانگین هندسی کوتینین سرم بندناف نوزاد در گروه مواجهه یافته (ng/ml 22/1±71/3) نسبت‌به گروه مواجهه نیافته (ng/ml 63/0±404/0) بیشتر بوده است (0001/0>P). همچنین بین خودگزارش‌دهی خانم باردار از مواجهه با دود سیگار با کوتینین سرم بندناف نوزاد همبستگی قوی وجود دارد (کاپا 98%) و ارتباط آماری معنی‌داری مشاهده شد (0001/0>P). گزارش خانم‌های باردار از مواجهه با دود سیگار با میزان کوتینین سرم بندناف دارای حساسیت 98%، ویژگی 100%، ارزش اخباری مثبت 100% و ارزش اخباری منفی 98% مي‌باشد. نتيجه‌گيري: این مطالعه ثابت نمود که خودگزارش‌دهی خانم باردار از مواجهه با دود سیگار در دوران بارداری از صحت بالایی برخوردار مي‌باشد و پیشنهاد مي‌کند ؛ چنانچه محتوای پرسشنامه ساختاری و تکنیک مصاحبه دقیق باشد مي‌توان از آن به‌عنوان جانشین قوی برای اندازه‌گيري بیومارکرهای شیمیایی دود سیگار نظیر کوتینین لحاظ نمود.

Title: بررسی نگرانی‌های مرتبط با بیماری در بیماران مبتلا به نارسایی قلبی

http://knh.shmu.ac.ir/index.php/site/article/view/356

مقدمه: نارسايی قلبی یک مشکل عمده رو به رشد است که نه تنها بیماران بلکه خانواده آنان و شبکه اجتماع را نیز تحت تأثير قرار می‌دهد و باعث کاهش ظرفیت‌های عملکردی بیماران و اختلال در زندگی اجتماعی افراد می‌گردد. پژوهش حاضر با هدف تعیین نگرانی‌های مرتبط با بیماری در بیماران مبتلا به نارسایی قلبی صورت گرفته است. مواد و روش‌ها: در مطالعه توصيفي- مقطعی حاضر، تعداد 130 بيمار مبتلا به کلاس II تا IV نارسایی قلبی بر اساس مشخصات موردنظر پژوهشگر و به‌صورت نمونه‏گيري دردسترس از بیمارستان‌های حضرت رسول (ص)، امام حسین (ع) و شریعتی تهران در سال 1391 انتخاب گرديدند، پرسشنامه اطلّاعات دموگرافيك و همچنين پرسشنامه نگرانی‌های مرتبط با بیماری IRWQ))، بر اساس گفته‌هاي بيمار تكميل گرديد. تجزیه‌و‌تحلیل آماری با استفاده از نرم‌افزار SPSS انجام شد. نتایج: بر اساس نتایج به‌دست آمده، میانگین سنی واحدهای مورد پژوهش 9/13±62 بود و اکثریت آنان (5/51 درصد) از نظر تحصیلات بی‌سواد بودند و درآمد متوسّط ماهانه كمتر از یک میلیون تومان در ماه (3/92 درصد) داشتند. میانگین نمره نگرانی‌های مرتبط با بیماری در واحدهای مورد پژوهش 1/1 ± 9/3 محاسبه گردید. بیشترین میانگین نمره، مربوط ‌‌به نگرانی از عوارض جسمی- روانی و کمترین میانگین نمره، مربوط‌به نگرانی از آینده بیماری بود. آزمون آماری آنالیز واریانس، تفاوت معناداری از لحاظ میانگین نمره نگرانی‌های‌ مرتبط با بیماری بین ابعاد مختلف و بین دو بعد: نگرانی از عوارض جسمی- روانی و نگرانی از آینده بیماری نشان داد (05/0P<). آزمون آماری پیرسون، ارتباط معنی‌دار آماری بین سن با نمره نگرانی‌های مرتبط با بیماری و بین دفعات بستری با نمره نگرانی از آینده بیماری نشان داد. نتیجه‌گیری: باتوجه به نتایج مطالعه، به‌كارگيري از راهکارهای کاهش نگرانی در بیماران مبتلا به نارسایی قلبی و برنامه‌ریزی جهت حمایت، درمان و مراقبت از اين بیماران و نيز انجام پژوهش‌های بعدی مرتبط با نگرانی‌های بیماران سودمند به‌نظر مي‌رسد.

Title: توسعه و طراحی راهنمای آموزشی خودمراقبتی براي بهبود علائم گرگرفتگی دوران یائسگی با تأكيد بر فعالیت جسمانی و رژیم غذایی در زنان 45-60 سال

http://knh.shmu.ac.ir/index.php/site/article/view/452

مقدمه: هدف از این مطالعه توسعه و تدوین راهنمای آموزشی خود مراقبتی براي بهبود علائم گرگرفتگی در دوران یائسگی با تأكيد بر فعالیت جسمانی و رژیم غذایی مناسب در زنان 60 – 45 سال است. مواد و روش‌ها: این مطالعه از نوع مطالعات ترکیبی دو مرحله‌ای است. در مرحله اول یک مطالعه مروری انجام شده و کلیه مقالات خارجی و داخلی که در ده سال اخیر به تأثير رژیم غذایی و فعالیت بدنی درکاهش گرگرفتگی پرداخته بودند، استخراج و نتایج آنها ثبت گردید در مرحله دوم به‌روش کیفی از طریق برگزاری پانل خبرگان نظر افراد خبره در مورد داده‌های گردآوری شده بررسی و مورد بحث و تجزیه‌و‌تحلیل قرار گرفت و نتایج نهایی پس از تأیید خبرگان در قالب کتابچه‌ای ارائه گردید. نتایج: 410 مقاله از منابع خارجی و داخلی بررسی گردید و با حذف مقالات تکراری و غیرمرتبط در مجموع نتایج 77 مقاله تحلیل شد که 40 مقاله مرتبط با تغذیه و 37 مقاله مربوط به تمرینات بدنی بوده است و نتایج این مطالعات پس از ارائه در پانل خبرگان و بحث پیرامون آنها، تأیید گردید که استفاده یکی از رژیم‌های غذایی حاوی فیتواستروژن‌ها از‌جمله عصاره گیاه گل ساعتی 60 قطره در روز،عصاره ویتاگنوس 40 قطره در روز، پودر عصاره آبی شیرین بیان 60 میلی‌گرم روزانه و مصرف روزانه 60 گرم پودر سویا توأم با انجام فعالیت‌های هوازی ازجمله پیاده‌روی، به‌مدت 45-30 دقیقه با شدت متوسط رو به بالا (5-3 روز در هفته) در کاهش علائم و عوارض گرگرفتگی زنان 60 – 45 مؤثر است. نتیجه‌گیری: نتایج نشان داد که فعالیت بدنی منظم توأم با رژیم غذایی حاوی عصاره گیاه گل ساعتی، عصاره ویتاگنوس، عصاره شیرین بیان، فیتواستروژن‌ها از‌جمله سویا، در کاهش علائم و عوارض گرگرفتگی دوران یائسگی مؤثر است.

Title: تأثیر مصرف مکمل سویا و تمرینات منظم هوازی در آب بر علائم جسمی و روانی سندرم پیش از قاعدگی دانشجویان دختر

http://knh.shmu.ac.ir/index.php/site/article/view/400

مقدمه: هدف از این مطالعه بررسی تأثیر هشت هفته مصرف مکمل سویا و تمرینات منظم هوازی در آب بر علائم جسمی و روانی سندرم پیش از قاعدگی (PMS:pre menstrual syndrome) دانشجویان دختر بود. مواد و روش‌ها: این پژوهش یک کارآزمایی نیمه تجربی یک سوکور در بین دانشجویان دختر خوابگاه‌های دانشگاه شاهرود بود. در این تحقیق از بین 67 دانشجوی دختر مبتلا به PMS از نوع اختلال شدید که حائز شرایط ورود به مطالعه نیز بودند، تعداد 40 نفر به‌صورت داوطلبانه و با پر کردن فرم رضایت‌نامه حاضر به همکاری با ما تا پایان تحقیق شدند. پس از انجام مرحله پیش آزمون و تکمیل پرسشنامه (PMS) آزمودنی‌ها به شرح ذيل به 4 گروه 10 نفري تقسیم شدند: گروه تمرینات هوازی كه فعالیت هوازی در آب، به‌مدت 8 هفته، هفته‌ای سه جلسه با شدت 55 تا 65 درصد بيشينه ضربان قلب را انجام مي‌دادند، گروه مصرف مکمل سویا (روزی یک عدد کپسول 500 میلی‌گرمي)، گروه مکمل سویا به همراه تمرینات هوازی و گروه مصرف كننده‌ي دارونما كه روزی یک عدد کپسول که شبیه کپسول سویا بود را مصرف مي‌كردند. در پایان هشت هفته در مرحله پس آزمون دوباره پرسشنامه PMS به‌طور دقیق از طرف آزمودنی‌ها تکمیل گردید. در تحلیل داده‌ها با استفاده از نرم‌افزار SPSS18 استفاده و با‌توجه به نرمال بودن داده‌ها از آزمون ANOVA دو طرفه استفاده گردید. نتایج: یافته‌ها نشان داد هشت هفته تمرینات هوازی در آب بر علائم کلی، جسمی و روانی PMS در گروه تمرین تأثیر معنی‌داري دارد همچنین هشت هفته مصرف سویا بر علائم کلی و روانی PMS در گروه مکمل سویا تأثیر معنی‌داری داشته ولی بر علائم جسمی تفاوت معنی‌داري ندارد همچنین هشت هفته مصرف توأم سویا و تمرین هوازی در آب به‌صورت ترکیبی موجب كاهش علائم کلی، جسمی و روانی PMS گرديده ليكن اين تفاوت معنی‌داری نبود. نتيجه‌گيري: با‌توجه به نتایج تحقیق به‌نظر مي‌رسد كه بيماران با علائم جسمی و روانی سندرم پیش از قاعدگی جهت کاهش يا درمان علائم PMS مي‌توانند بسته به تمايل مصرف سويا و امكان تمرين در اب از مكمل سويا يا انجام ورزش‌هاي هوازي در آب استفاده نمايند.

Title: تأثير درمان واریکوسل بر شاخص‌های آنالیز اسپرم

http://knh.shmu.ac.ir/index.php/site/article/view/395

مقدمه: واریکوسل یکی از شایع‌ترین علل ناباروری مردانه قابل اصلاح بوده که به دلايل گوناگونی همواره تشخیص به‌موقع آن با تأخیر صورت مي‌گیرد. شیوع نسبتاً بالای واریکوسل در مردان، همواره تشخیص و درمان این مشکل را جزء اقدامات مهم ارولوژیک قرار داده است. مطالعات گوناگونی در‌خصوص درمان واریکوسل و تغییرات کیفیت مایع منی صورت گرفته که معمولاً به‌صورت کامل نبوده و نتایج متفاوتی داشته‌اند، لذا باتوجه به شیوع بالای واریکوسل در مردان و درمان نسبتاً ساده آن، برآن شدیم تا تغییرات مایع مني و کیفیت اسپرم را پس از عمل واریکوسلکتومی بررسی نمايیم. مواد و روش‌ها: این پژوهش به‌صورت مقطعی، برروي کلیه مراجعین داوطلب با تشخیص واریکوسل از سال 88 لغایت 90 صورت گرفت. در این تحقیق پس از تکمیل پرسشنامه خود تنظیمی برای هر بیمار، بررسی مایع اسپرم قبل و بعد از عمل (در دو نوبت سه و شش ماه پس از عمل) انجام شده و نتایج با هم مقایسه شد. نتايج: در این مطالعه میانگین سنی 250 نفر مراجعه‌کننده 9/3±2/35 سال بود. اكثر افراد تحت بررسي در گروه سني 31-40 سال (8/70%) بودند. از کل مراجعین 198 نفر (2/79%) متأهل بودند. درخصوص سمت درگیری واریکوسل، در 218 مورد (2/87%) سمت چپ درگیر بود. نتایج این پژوهش نشان داد که کلیه شاخص‌های اسپرم با عمل واریکوسلکتومی وضعیت بهتر و مطلوب‌تری پیدا نموده به‌طوری‌که بین شاخص‌های اسپرم مثل افزایش میانگین حجم مایع سیمن (001/0P<)، افزایش میانگین تعداد اسپرم (001/0P<)، افزایش تحرک اسپرم (001/0P=)، بهتر شدن مورفولوژی اسپرم (001/0P<) و اصلاح pH مایع مني (001/0P<) و عمل واریکوسلكتومی رابطه معناداری وجود داشت. نتيجه‌گيري: پژوهش حاصل نشان داد با تشخیص به‌موقع و درمان واریکوسل می‌توان تا حدود زیادی مشکلات موجود در کیفیت شاخص‌های اسپرم مردان را بهبود بخشیده و احتمالاً میزان ناباروری آنها را کاهش داد.
